# A Guiding Evolutionary Algorithm with Greedy Strategy for Global Optimization Problems

**DOI:** 10.1155/2016/2565809

**Published:** 2016-05-18

**Authors:** Leilei Cao, Lihong Xu, Erik D. Goodman

**Affiliations:** ^1^Department of Control Science and Engineering, Tongji University, Shanghai 201804, China; ^2^BEACON Center for the Study of Evolution in Action, Michigan State University, East Lansing, MI 48824, USA

## Abstract

A Guiding Evolutionary Algorithm (GEA) with greedy strategy for global optimization problems is proposed. Inspired by Particle Swarm Optimization, the Genetic Algorithm, and the Bat Algorithm, the GEA was designed to retain some advantages of each method while avoiding some disadvantages. In contrast to the usual Genetic Algorithm, each individual in GEA is crossed with the current global best one instead of a randomly selected individual. The current best individual served as a guide to attract offspring to its region of genotype space. Mutation was added to offspring according to a dynamic mutation probability. To increase the capability of exploitation, a local search mechanism was applied to new individuals according to a dynamic probability of local search. Experimental results show that GEA outperformed the other three typical global optimization algorithms with which it was compared.

## 1. Introduction

Most real-world optimization problems in engineering, management, and other applications are of high dimensionality, multimodal, and/or multiobjective. Methods for solving these problems range from classical mathematical methods to heuristic algorithms. With more complex problems, heuristic intelligent algorithms have been used successfully and frequently [1]. These algorithms include Simulated Annealing [2], Genetic Algorithm [3], Particle Swarm Optimization [4], Ant Colony Optimization [5], Artificial Bee Colony [6], Firefly Algorithm [7, 8], and Bat Algorithm [9]. Support for parallel computation and stochastic search are common characteristics of these algorithms. Inevitably, most of these algorithms are slow to converge or converge prematurely, trapped in local optima upon addressing high-dimensional or multimodal optimization problems [10]. High-dimensional variables expand the search space, influencing the required number of evaluations and speed of convergence. Multimodality means that there exist one or more global optima and some number of locally optimal values [10, 11]. The “No Free Lunch” theorem clarifies that the performance of any search algorithm depends on the complexity of the problem domain [12].

All of the above-mentioned heuristic algorithms are population-based except Simulated Annealing, and some of them are classified as swarm intelligence. These population-based algorithms, when applied to complex problems, frequently outperform classical methods such as linear programming [13], gradient methods [14], and greedy algorithms [15]. Although the heuristic algorithms can solve some problems, sometimes they converge on local optima or find solutions near but not at the global optimum.

This paper introduces a Guiding Evolutionary Algorithm to address global optimization problems. It is inspired by Particle Swarm Optimization and the Bat Algorithm and uses some methods from the Genetic Algorithm. It is easily understood and implemented. For some problem classes tested, this algorithm has outperformed canonical implementation of those individual heuristic methods. This paper is organized as follows: Section 2 gives brief overview about PSO and BA.  Section 3 proposes this new global optimization algorithm. A set of test functions and the experimental results are presented in Section 4, and conclusions are presented in Section 5.

## 2. Particle Swarm Optimization and Bat Algorithm

### 2.1. Overview of PSO

A variety of heuristic algorithms are called swarm intelligence algorithms, simulating collaborative behavior of a swarm of insects, birds, fish, or other animals searching for food [4, 16]. Among them, Particle Swarm Optimization (PSO) is the most widely used. PSO was first proposed by Eberhart and Kennedy [4] in 1995. Each solution in PSO is a “particle” that searches in the independent variable space by learning from its own experience and other particles' past experiences [17, 18]. There are two different ways of representing others' past experiences: the global best and the local best. In global optimization problems, each particle adjusts its velocity and position according to the historical best position of itself and the best position among all particles. But this method tends to make each particle converge to a local optimum in those problems containing many locally optimal values. For these problems, the local best method is more effective. Each particle adjusts its velocity and position according to its historical best position and the best solution found within its neighborhood. In fact, if the neighborhood is all particles, the local best becomes the global best version. PSO is simple and easy to be implemented, but PSO lacks a mutation mechanism which could increase the diversity of the population and help to avoid convergence at local optima.

### 2.2. Bat Algorithm [9, 19–21]

The Bat Algorithm (BA) was proposed by Yang in 2010. It is based on the echolocation behavior of bats, which can find their prey and discriminate different types of insects even in complete darkness. This algorithm combines global search with local search in each iteration, which balances exploration and exploitation during search. The algorithm defines the rules for how these bats' positions and velocities in a *d*-dimensional search space are updated. The new positions and velocities at time step *t* are given by

(1) fi=fmin+fmax−fmin∗β,vit=vit−1+xit−1−x∗t−1∗fi,xit=xit−1+vit,

where *β* ∈ [0,1] is a random vector and *x*_*∗*_^*t*−1^ is the global best location found to date. *f*_*i*_ is the frequency of the wave.

For the local search part, a new solution for each bat is generated locally using a random walk around the current best solution:

(2) $$\begin{array}{c} x_{new}=x_{old}+\varepsilon *A^{t}, \end{array}$$

where *ε* ∈ [−1,1] is a uniformly distributed random number and *A* ^*t*^ is the average loudness of all bats at this time step.

The update of the velocities and positions of bats has some similarity to the procedure in the standard Particle Swarm Optimization as *f*_*i*_ essentially controls the pace and range of movement of the swarming particles. To a degree, BA can be considered as a balanced combination of the standard Particle Swarm Optimization and an intensive local search controlled by the loudness and pulse rate. Furthermore, the loudness *A*_*i*_ and rate *r*_*i*_ of pulse emission must be updated accordingly as the iterations proceed. The loudness usually decreases once a bat has found its prey, while the rate increases.

## 3. A Guiding Evolutionary Algorithm

Although Bat Algorithm can solve some difficult problems and converge quickly, it often cannot avoid converging to a local optimum, especially for those high-dimensional multimodal problems. Examining PSO and BA reveals some differences, in that BA rejects the historical experience of each individual's own position but accepts a better personal solution with some probability. We will modify some of the updating mechanisms of BA and add a mutation method in order to try to address global optimization problems more accurately.

### 3.1. Crossover

There is a leading individual in both PSO and BA, which can lead all individuals to update their own positions. But we find that current velocities of individuals are weighted accumulations of their own historical velocities across iterations. In the later parts of a search, this will tend to decrease the speed of convergence or favor convergence on local optima, even though there are bounds on velocities in PSO. To avoid these situations and employ the leading individual effectively, we use the following expression:

(3) $$\begin{array}{c} x_{i}^{t}=x_{i}^{t-1}+\left(\;x_{*}^{t-1}-x_{i}^{t-1}\;\right)*\beta , \end{array}$$

where *x*_*∗*_^*t*−1^ is the current global best individual and *β* is the step length of the position increment.

**Figure 1:**
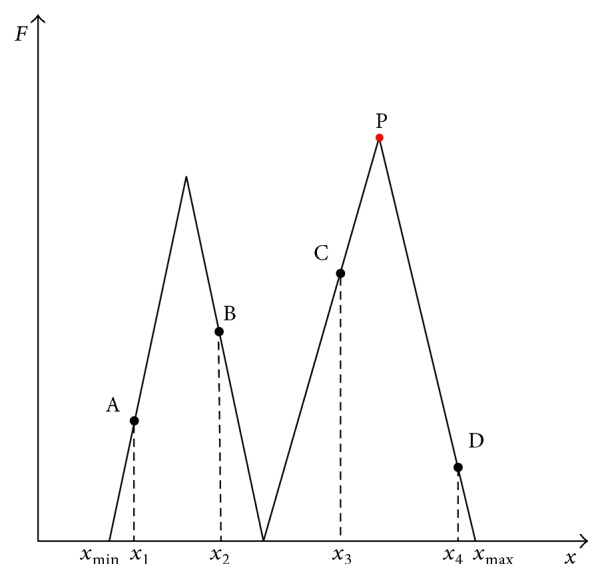
A two peaks' function.

In fact, this expression is a form of crossover between each individual and the best individual to generate their offspring. *x*_*∗*_^*t*−1^ is a guide to lead all other individuals to walk forward toward the current best one. To explain formula (3) clearly, we use a simple example to describe the process of locating the global best individual.  Figure 1 shows a two peaks' function, the ordinate of which is the value of the objective function and the abscissa of which is the design variable. Point P is the global best we expect to find in this maximization problem. Points A, B, C, and D indicate four individuals, and *x*_1_, *x*_2_, *x*_3_, and *x*_4_ are their solutions in variable space, respectively. *x*_min_ and *x*_max_ are the bounds of the variable space. Among the four individuals, point C is the best; therefore, other individuals will move toward this current best individual. Let us assume *β* ∈ [0,2] is a uniform random variable. For point A, its motion ranges between *x*_1_ and *x*_max_, because 2*∗*(*x*_3_ − *x*_1_) exceeds the upper bound of the variable range. Theoretically, point A can move to each point whose variable value is between *x*_1_ and *x*_max_, and this will depend on the random variable *β*. Not only point A but also points B and D will be attracted by point C. The best individual will be replaced once a better solution occurs. After many iterations, all of these individuals can reach the global best point P. While searching, even though the best individual may reach the lower peak, other individuals can also move to point P because of random walking and a great number of population.

### 3.2. Mutation

**Figure 2:**
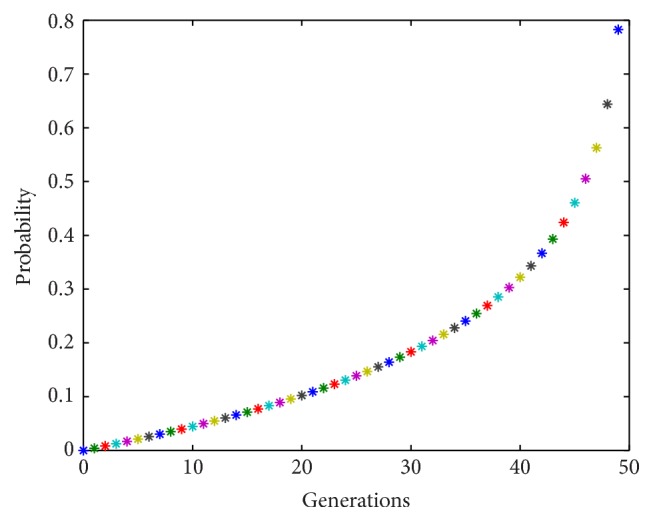
Probability of mutation.

Although these global optimization algorithms work quite well on some engineering problems, they sometimes cannot avoid converging on a local optimum with high probability. For such heuristic algorithms, it is difficult to prove that they will always converge to a global optimum. In order to raise the probability of converging to a global optimum, we add a mutation mechanism which is inspired by the Genetic Algorithm. The purpose of mutation is to increase the diversity of the population and prevent them trapping into a local optimum, especially in the later iterations. Therefore, the probability of mutation will be made low at the beginning and higher later. We set the mutation probability as follows:

(4) $$\begin{array}{c} p=c*\ln\left(\;\frac{T_{max}}{T_{max}-t}\;\right), \end{array}$$

where *c* is a limiting parameter which can be a constant or a variable, *T*_max_ is the maximum number of generations, and *t* is the current generation. We will assume that *T*_max_ = 50 and *c* = 0.2; then, probability *p* increases with iterations as shown in Figure 2. Especially in the early iterations, *p* is low, while it increases rapidly in the later iterations.

The mutation formula is as follows:

(5) $$\begin{array}{c} x_{i}^{t}=x_{i}^{t}+\varepsilon *M, \end{array}$$

where *x*_*i*_^*t*^ is the solution of an individual after crossover, *ε* ∈ [−1,1] is a uniform random number, and *M* is a vector which determines the scope of mutation. In general, we set *M* such that mutation can span the entire range of the independent variables. We will assume that the range of *j*th dimension of the variables is [*a*, *b*], and then

(6) $$\begin{array}{c} M_{j}=\max\left(\;x_{ij}^{t}-a,b-x_{ij}^{t}\;\right). \end{array}$$

Each dimension of the vector *M* is set as formula (6). Sometimes the solution after mutation will exceed the range of the variables, and we can limit it to the bounds in this situation.

### 3.3. Local Search

As we know, most of global optimization algorithms have excellent capability of exploration but are weak at exploitation. To enhance this ability, especially in the later iterations, we will expect the algorithm to be able to locate the global best quickly with local search, once it has found the right neighborhood. The probability of local search will be maintained low in early iterations and raised later in the search process. The probability of local search will follow the same distribution as mutation (4).

Each individual has the calculated probability of being replaced by a result of local search, performed around the current best individual. Similarly to the Bat Algorithm's random walk, we use the following formula to express local search:

(7) $$\begin{array}{c} x_{i}^{t}=x_{*}^{t-1}+\varepsilon *L, \end{array}$$

where *x*_*∗*_^*t*−1^ is the best individual of the current population, *ε* ∈ [−1,1] is a uniform random number, and *L* is a vector which determines the search scope of the random walk, formulated in the variable space. We still will assume that the range of *j*th dimension of the variables is [*a*, *b*], and then

(8) $$\begin{array}{c} L_{j}=\text{10\%}*\left(\;b-a\;\right). \end{array}$$

10% is only an example of scope of the random walk. The other dimensions of the vector *L* are set as formula (8). Local search acts much like mutation; the differences are in the points to which the mutational changes are added—the global best in one case and any individual in the other.

### 3.4. Summary of GEA

Unlike the classical (greedy) simplex algorithm, a population-based algorithm can “jump out” of a local optimum using parallel search, without losing all prior information. Therefore, we can afford to accept a new global best-to-date individual when it is found, instead of accepting it according to some probability, as Simulated Annealing must do in order to reduce getting trapped at local optima. In the GEA, each individual updates only when its offspring has better fitness. The updates of the global best individual and each individual are greedy strategies, but, because of a large population size, convergence to local optima can be avoided in some multimodal global optimization problems.

The proposed Guiding Evolutionary Algorithm with greedy strategy resembles the framework of the Genetic Algorithm, which can be described as follows: initialization, evaluation, selection, crossover, and mutation. A difference from the Genetic Algorithm is that there is no special selection mechanism in the GEA, because each individual will generate its offspring by recombination with the global best individual and it does not require an operator to select an individual to evolve. In addition, local search is also employed to increase the algorithm's exploitation capability. The whole framework is easy to understand and implement. The whole pseudocode of this algorithm is as shown in Algorithm 1.

## 4. Experimental Results and Discussion

### 4.1. Test Functions [11, 22, 23]

In order to test the proposed algorithm, 6 commonly used functions are chosen. *F*_1_–*F*_6_ are well-known test functions for global optimization algorithms including Genetic Algorithm, Particle Swarm Optimization, Bat Algorithm, Firework Algorithm [24], and Flower Pollination Algorithm [25]. *F*_1_ is a simple unimodal and convex function; *F*_2_ is a unimodal but nonconvex function; *F*_3_ is multimodal, since the number of local optima increases with the dimensionality; *F*_4_ has a global minimum at 0, which is inside the parabolic, narrow-shaped flat valley (variables are strongly dependent on each other; therefore it is difficult to converge on the global optimum); *F*_5_ is multimodal and has many local minima but only one global minimum; *F*_6_ also has numerous local minima and is multimodal. The definitions of these benchmark functions are listed in Table 1. All of their global minima are at 0.

### 4.2. Parameter Settings for Testing Algorithms

In this section, the Guiding Evolutionary Algorithm (GEA), Fast Evolutionary Algorithm (FEA) [23], Particle Swarm Optimization (PSO), and Bat Algorithm (BA) are tested separately on six benchmark functions. For *F*_1_ to *F*_6_, to illustrate how the dimensionality of problems influences the performance of the algorithms, three different dimensions of benchmark functions are used: *D* = 10, *D* = 20, and *D* = 30. The parameters of the Fast Evolutionary Algorithm are set as in [23]. Through some trials, we set the number of generations in FEA, PSO, and BA as 500, while the number of generations is set as 50 in GEA, in order to get the best results. Population sizes were adjusted according to the different benchmark functions and different dimensions. Although these algorithms run for different numbers of generations, the numbers of evaluations are the same for any given benchmark function. For PSO, according to some researches [17, 18], the following settings can make it perform well: the inertia weight is set as 0.7, while the two learning parameters are set as 1.5; the limitation of velocity is set as [−1, 1]. Parameters of BA are set as follows: minimum frequency *f*_min_ = 0, maximum frequency *f*_max_ = 2.0, the loudness *A*_0_ = 0.8, the pulse rate *r*_0_ = 0.8, and the control factor *α* = *β* = 0.9. These parameters are attributed to some published articles [11, 19]. For our GEA, *β* ∈ [0,2] and *c* = 0.2.

Generally speaking, for unimodal functions, the convergence rate is of primary interest, as optimizing such functions to a satisfactory accuracy is not a major issue. For multimodal functions, the quality of final solutions is more significant since they can illustrate the ability of the algorithm tested, to escape from local optima and locate the true global optimum. Based on these requirements, we present the computational optimal solutions of these benchmarks using our GEA as well as comparing with other algorithms. In addition, the curves of convergence rate are plotted in order to indicate the numbers of evaluations to reach the approximate global optimum or converge on a local optimum.

### 4.3. Experimental Results and Discussion

#### 4.3.1. Results for the 10D Benchmark Functions

**Figure 3:**
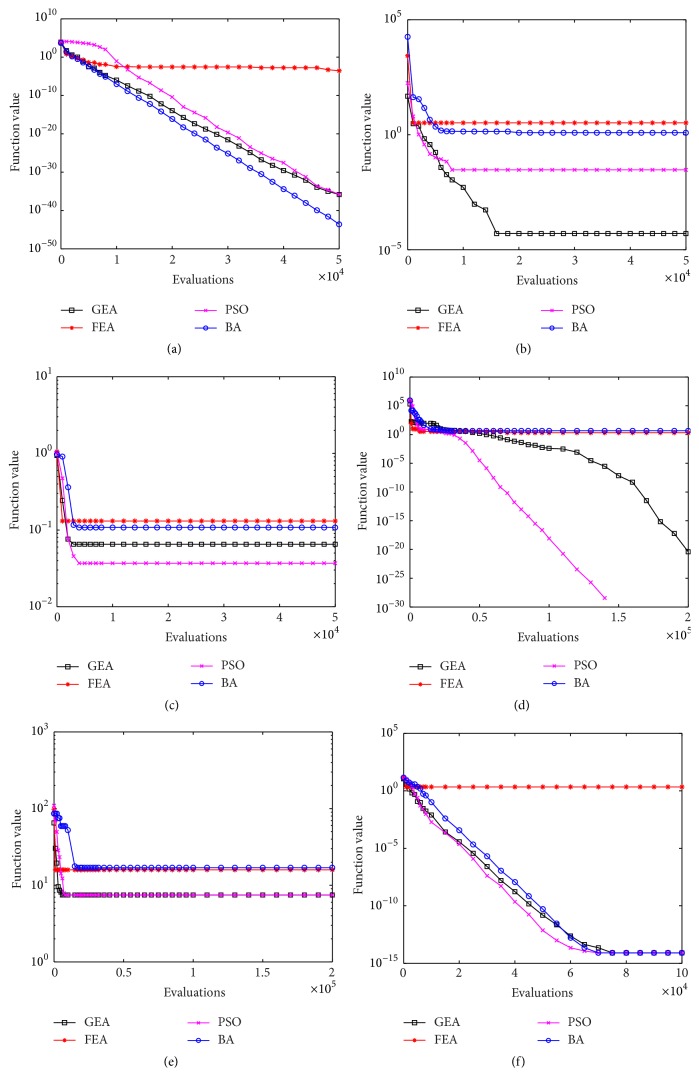
The median value convergence characteristics of 10D benchmark functions. (a) *F*_1_; (b) *F*_2_; (c) *F*_3_; (d) *F*_4_; (e) *F*_5_; and (f) *F*_6_.

Table 2 depicts the computational results of four algorithms on six benchmark functions with 10 dimensions, which were run 20 times independently to get the best, worst, mean, median, and standard deviation values. Among these five values, the “median” item can indicate the algorithm's optimizing performance reasonably, while the “standard deviation” item denotes the stability of the algorithm's optimizing performance.  Figure 3 presents the convergence characteristics in terms of median value of 20 runs of each algorithm on each benchmark function.

From the computational results, we find that BA performs best among these four algorithms on *F*_1_, although three of them have excellent performances. As stated earlier, the convergence rate is more crucial in comparison with convergence accuracy for unimodal functions. As indicated from Figure 3(a), GEA, PSO, and BA have fast convergence rate and high convergence accuracy, but FEA gets the worst accuracy. Actually, *F*_1_ is only a simple unimodal and convex function. There is no mutation operation in the BA's procedure but only local search. However, compared with GEA and PSO, the local search and absence of mutation become the BA's advantage in addressing unimodal and convex functions. But this advantage is lose upon testing unimodal and nonconvex function *F*_2_. GEA clearly outperforms the other algorithms in convergence rate and convergence accuracy; the median and mean value are close to the true global optimum. Viewed from Figure 3(b), it is easy to see that GEA and PSO have similar search processes in the early evaluations, but GEA can jump out from a local optimum because of its mutation operation and continue to search with local search, while PSO stops at the local optimum. GEA improves several orders of magnitude compared to the results of the other algorithms. In addition, it has better stability of optimization performance. *F*_3_ is a multimodal function; both GEA and PSO perform well, and they have similar convergence rates and accuracies as indicated from Figure 3(c). We display the ability to avoid falling into local optima. As described, function *F*_4_ poses special difficulties for an optimizer. Although PSO locates the minimization value exactly in median value on *F*_4_, PSO's stability is worse than that of GEA. Sometimes, locating the exact optimum is not our purpose, but rather the stable computational performance of the algorithm is more significant. Each of GEA's values on *F*_4_ is close to the real global optimum, and the stability of its optimizing performance is excellent. This performance benefits from changeable mutation parameters and local search areas. But, as viewed from Figure 3(d), the convergence accuracies of FEA and BA are worse than those of GEA and PSO. *F*_5_ and *F*_6_ are multimodal functions, but *F*_5_ makes it extremely difficult to locate the global optimum, while *F*_6_ is much easier. GEA and PSO have similar performance upon addressing *F*_5_ and *F*_6_, while GEA is a little better than PSO especially in standard deviation. Although neither of them can locate the real global minimization on *F*_5_, they still work better than FEA and BA. As indicated from Figure 3(e), although FEA has the fastest convergence rate, the convergence accuracy is too poor similar to that of BA. In addition, BA also performs well upon addressing *F*_6_, but it is not yet better than GEA and PSO. Their median values are very close to the real global minimum. The convergence rates of GEA, PSO, and BA are also similar, but FEA has been trapped in a local optimum prematurely as seen from Figure 3(f).

#### 4.3.2. Results for the 20D and 30D Benchmark Functions

Table 3 shows these four algorithms' computational results on six 20D benchmark functions, and Table 4 shows computational results on six 30D benchmark functions. As the convergence graphs are similar to those of the 10D functions except the convergence accuracy, they are not presented here. It is easy to see that increasing the dimension of *F*_1_ did not influence the nature of the performance of these algorithms, just increasing the number of evaluations needed to get similar accuracy. Increased dimension of *F*_2_ reduces the convergence accuracy of GEA, PSO, and BA, but FEA is not affected by that because FEA can never get good accuracy. Sometimes we can accept the results of GEA and PSO on *F*_2_ in 20D and 30D, but that of FEA and BA cannot be accepted. The effects of *F*_3_ on 20D and 30D are better than those on 10D for these four algorithms. This problem is known to become easier as the dimension increases [26]. GEA performs best among the methods on *F*_3_, although increasing the problem's dimension and its convergence accuracy brings the computational optimum closer to the real global optimum for all tested methods. For *F*_4_, *F*_5_, and *F*_6_, with increasing dimensions, none of these algorithms can locate the real global optimum or approach it closely. But GEA is the best compared with the other algorithms in optimization results. High dimension is a calamity for these problems, which makes it difficult to locate their real global optimum.

#### 4.3.3. Discussion

Analysis of the results of four algorithms on six functions with different dimensions indicates that GEA has better optimization performance compared to FEA, PSO, and BA. BA is suitable to address simple unimodal problems, but it has an undistinguished performance on other problems, especially on multimodal problems. FEA has a fast convergence rate, but its convergence accuracy is too bad in some problems. Sometimes GEA and PSO have similar computational results and convergence rate and accuracy on multimodal problems, but GEA performs more stably because of its changeable mutation operation and local search, which can increase its capability of exploration and exploitation. The changeable mutation operation will reduce the convergence rate, but the greedy strategy offsets this reduction. Therefore, GEA has a convergence rate as quick as that of PSO and BA, and the convergence accuracy of GEA is better than/or similar to that of the others.

## 5. Conclusion

In this paper, a Guiding Evolutionary Algorithm (GEA) has been proposed to solve global optimization problems. This algorithm mixes advantage of Particle Swarm Optimization, Bat Algorithm, and Genetic Algorithm. The largest difference is that the GEA accepts a newly generated individual only when its fitness is better than that of the parent, while the PSO and many versions of the GA always accept the new individual. The BA accepts the better solution according to some given probability. This mechanism of GEA can guarantee that each individual stays in its historically best position and moves forward toward the global best position found to date. Comparing with PSO and BA, the velocity was removed from GEA but replaced by a random walk step toward the current global best individual. After crossover with the global best individual, mutation was added to the generated individual according to a mutation probability. To increase the algorithm's exploitation power, a local search mechanism was applied to a new individual according to a given probability of local search; this mechanism actually produces a random walk around the current global best individual.

Experimental results show that GEA can approximate the globally optimal solution very accurately in most of the problems tested. Comparing with three other typical global optimization algorithms, GEA performs more accurately and stably. In the future work, niching methods will be considered for addition to the GEA to solve those multimodal problems yet more effectively.

## Figures and Tables

**Algorithm 1:**
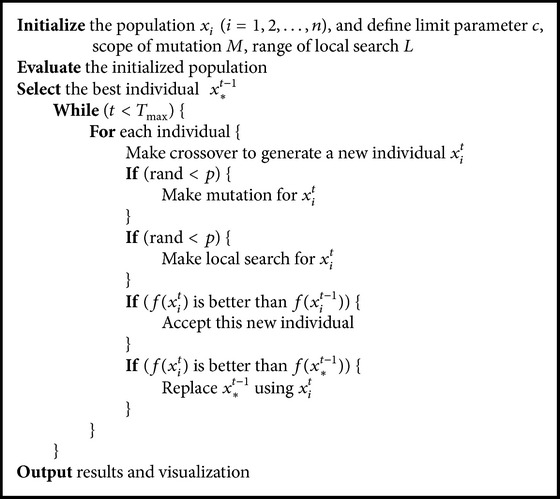


**Table 1:** Six test functions utilized in this experiment.

Functions	Name of function	Expression	Domain of variables
*F*_1_	De Jong's sphere function	$f\left(x\right)=\overset{D}{\underset{i=1}{\sum }}x_{i}^{2}$	[−100,100]

*F*_2_	Schwefel 2.22 function	$f\left(x\right)=\overset{D}{\underset{i=1}{\sum }}\left|x_{i}\right|+\prod_{i=1}^{D}\left|x_{i}\right|$	[−15,15]

*F*_3_	Griewangk's function	$f\left(x\right)=-\prod_{i=1}^{D}\cos\left(\frac{x_{i}}{\sqrt{i}}\right)+\overset{D}{\underset{i=1}{\sum }}\frac{x_{i}^{2}}{4000}+1$	[−15,15]

*F*_4_	Rosenbrock's function	$f\left(x\right)=\overset{D-1}{\underset{i=1}{\sum }}100*{\left(x_{i+1}-x_{i}^{2}\right)}^{2}+{\left(x_{i}-1\right)}^{2}$	[−15,15]

*F*_5_	Rastrigin's function	$f\left(x\right)=D*10+\overset{D}{\underset{i=1}{\sum }}\left(x_{i}^{2}-10*\cos\left(2\pi x_{i}\right)\right)$	[−5,5]

*F*_6_	Ackley's function	$f\left(x\right)=20+e-20*\exp\left(-0.2*\sqrt{\frac{1}{D}*\overset{D}{\underset{i=1}{\sum }}x_{i}^{2}}\right)-\exp\left(\frac{1}{D}*\overset{D}{\underset{i=1}{\sum }}\cos\left(2\pi x_{i}^{2}\right)\right)$	[−15,15]

**Table 2:** Results of benchmark functions in 10 dimensions using four algorithms.

Functions	Evaluations	Value	GEA	FEA	PSO	BA
*F*_1_	50000	Best	3.45*E* − 37	2.31*E* − 15	5.42*E* − 38	1.20**E** − 44
		Worst	6.62*E* − 35	1.95*E* − 03	1.05*E* − 34	5.57**E** − 44
		Mean	5.86*E* − 36	4.83*E* − 04	1.34*E* − 35	2.41**E** − 44
		Median	1.44*E* − 36	2.89*E* − 04	1.48*E* − 36	2.37**E** − 44
		StDev	1.46*E* − 35	5.98*E* − 04	2.88*E* − 35	1.05**E** − 44

*F*_2_	50000	Best	5.25*E* − 11	1.23*E* + 00	3.79*E* − 04	3.74**E** − 22
		Worst	3.44**E** − 04	7.27*E* + 00	2.11*E* − 01	5.11*E* + 01
		Mean	1.28**E** − 04	3.89*E* + 00	6.86*E* − 02	7.84*E* + 00
		Median	5.03**E** − 05	3.28*E* + 00	2.97*E* − 02	1.23*E* + 00
		StDev	1.20**E** − 04	1.96*E* + 00	8.52*E* − 02	1.42*E* + 01

*F*_3_	50000	Best	2.46*E* − 02	2.34*E* − 02	7.40**E** − 03	2.46*E* − 02
		Worst	1.06**E** − 01	3.37*E* − 01	1.62*E* − 01	2.63*E* − 01
		Mean	6.37*E* − 02	1.36*E* − 01	4.88**E** − 02	1.24*E* − 01
		Median	6.52*E* − 02	1.31*E* − 01	3.69**E** − 02	1.08*E* − 01
		StDev	2.39**E** − 02	7.12*E* − 02	3.82*E* − 02	6.86*E* − 02

*F*_4_	200000	Best	4.23*E* − 29	4.60*E* − 03	0**E** + 00	1.78*E* + 00
		Worst	6.91**E** − 10	6.16*E* + 00	3.99*E* + 00	6.98*E* + 00
		Mean	3.52**E** − 11	2.99*E* + 00	5.98*E* − 01	4.44*E* + 00
		Median	3.88*E* − 21	2.04*E* + 00	0**E** + 00	4.43*E* + 00
		StDev	1.54**E** − 10	2.55*E* + 00	1.46*E* + 00	1.31*E* + 00

*F*_5_	200000	Best	4.97*E* + 00	6.96*E* + 00	3.98**E** + 00	6.96*E* + 00
		Worst	1.19**E** + 01	2.69*E* + 01	1.39*E* + 01	2.69*E* + 01
		Mean	7.56**E** + 00	1.71*E* + 01	7.71*E* + 00	1.77*E* + 01
		Median	7.46**E** + 00	1.59*E* + 01	7.46*E* + 00	1.69*E* + 01
		StDev	1.69**E** + 00	5.82*E* + 00	2.77*E* + 00	4.82*E* + 00

*F*_6_	100000	Best	7.99**E** − 15	1.16*E* + 00	7.99*E* − 15	7.99*E* − 15
		Worst	1.65**E** + 00	3.40*E* + 00	1.65*E* + 00	2.32*E* + 00
		Mean	1.40**E** − 01	2.11*E* + 00	3.96*E* − 01	7.13*E* − 01
		Median	7.99**E** − 15	2.17*E* + 00	7.99*E* − 15	7.99*E* − 15
		StDev	4.38**E** − 01	7.93*E* − 01	6.34*E* − 01	9.39*E* − 01

**Table 3:** Results of benchmark functions in 20 dimensions using four algorithms.

Functions	Evaluations	Value	GEA	FEA	PSO	BA
*F*_1_	200000	Best	1.56*E* − 36	2.26*E* − 09	4.59*E* − 30	4.07**E** − 44
		Worst	4.72*E* − 35	6.20*E* − 04	5.30*E* − 15	1.18**E** − 43
		Mean	1.30*E* − 35	1.84*E* − 04	3.56*E* − 16	7.51**E** − 44
		Median	7.39*E* − 36	9.57*E* − 05	4.45*E* − 26	7.40**E** − 44
		StDev	1.37*E* − 35	2.02*E* − 04	1.22*E* − 15	2.06**E** − 44

*F*_2_	200000	Best	4.23*E* − 03	1.24*E* + 00	3.57*E* − 03	8.71**E** − 22
		Worst	3.07**E** − 01	4.32*E* + 00	1.36*E* + 00	1.31*E* + 02
		Mean	6.83**E** − 02	3.11*E* + 00	2.29*E* − 01	6.54*E* + 01
		Median	5.30**E** − 02	3.13*E* + 00	1.10*E* − 01	7.71*E* + 01
		StDev	6.86**E** − 02	9.24*E* − 01	3.32*E* − 01	4.39*E* + 01

*F*_3_	200000	Best	0**E** + 00	1.95*E* − 03	0*E* + 00	1.11*E* − 16
		Worst	7.40**E** − 03	8.13*E* − 02	1.23*E* − 02	7.13*E* − 02
		Mean	1.85**E** − 03	2.74*E* − 02	2.22*E* − 03	2.93*E* − 02
		Median	1.11**E** − 16	2.30*E* − 02	1.67*E* − 16	2.22*E* − 02
		StDev	3.29**E** − 03	1.92*E* − 02	4.07*E* − 03	2.19*E* − 02

*F*_4_	400000	Best	3.20**E** + 00	2.21*E* − 02	5.33*E* + 00	1.39*E* + 01
		Worst	1.26**E** + 01	4.93*E* + 01	1.69*E* + 01	1.51*E* + 02
		Mean	8.90**E** + 00	1.80*E* + 01	1.12*E* + 01	3.92*E* + 01
		Median	9.34**E** + 00	1.90*E* + 01	1.16*E* + 01	1.59*E* + 01
		StDev	2.00**E** + 00	9.30*E* + 00	2.56*E* + 00	4.58*E* + 01

*F*_5_	400000	Best	7.96**E** + 00	1.29*E* + 01	7.96*E* + 00	1.68*E* + 02
		Worst	2.39**E** + 01	8.56*E* + 01	2.89*E* + 01	2.35*E* + 02
		Mean	1.79*E* + 01	4.55*E* + 01	1.66**E** + 01	1.93*E* + 02
		Median	1.89*E* + 01	4.58*E* + 01	1.64**E** + 01	1.92*E* + 02
		StDev	3.73**E** + 00	2.04*E* + 01	6.29*E* + 00	1.67*E* + 01

*F*_6_	200000	Best	1.51**E** − 14	1.18*E* + 00	1.51*E* − 14	1.33*E* + 01
		Worst	2.17**E** + 00	3.95*E* + 00	2.45*E* + 00	1.54*E* + 01
		Mean	8.92**E** − 01	2.95*E* + 00	9.60*E* − 01	1.44*E* + 01
		Median	1.16**E** + 00	3.04*E* + 00	1.16*E* + 00	1.45*E* + 01
		StDev	7.86*E* − 01	6.62**E** − 01	7.89*E* − 01	6.77*E* − 01

**Table 4:** Results of benchmark functions in 30 dimensions using four algorithms.

Functions	Evaluations	Value	GEA	FEA	PSO	BA
*F*_1_	400000	Best	9.32*E* − 35	1.22*E* − 08	6.14*E* − 12	1.06**E** − 43
		Worst	6.01*E* − 33	3.86*E* − 04	2.15*E* − 07	2.42**E** − 43
		Mean	1.28*E* − 33	5.09*E* − 05	1.99*E* − 08	1.62**E** − 43
		Median	6.57*E* − 34	1.86*E* − 05	8.45*E* − 10	1.62**E** − 43
		StDev	1.467*E* − 33	8.90*E* − 05	5.01*E* − 08	3.41**E** − 44

*F*_2_	400000	Best	2.18*E* − 02	1.73*E* + 00	7.29*E* − 01	9.46**E** − 22
		Worst	6.98**E** − 01	5.65*E* + 00	9.51*E* − 01	1.93*E* + 02
		Mean	2.66**E** − 01	3.37*E* + 00	3.90*E* − 01	7.45*E* + 01
		Median	2.35**E** − 01	3.30*E* + 00	3.21*E* − 01	6.32*E* + 01
		StDev	1.89**E** − 01	1.11*E* + 00	2.45*E* − 01	7.04*E* + 01

*F*_3_	400000	Best	8.88**E** − 15	2.76*E* − 02	4.68*E* − 12	1.11*E* − 16
		Worst	9.86**E** − 03	1.43*E* − 01	9.88*E* − 03	2.71*E* − 02
		Mean	1.36**E** − 03	7.59*E* − 02	2.10*E* − 03	8.99*E* − 03
		Median	2.98**E** − 13	6.67*E* − 02	6.39*E* − 06	8.63*E* − 03
		StDev	3.34**E** − 03	3.53*E* − 02	3.77*E* − 03	7.79*E* − 03

*F*_4_	600000	Best	1.37**E** + 01	2.90*E* + 00	2.01*E* + 01	1.92*E* + 01
		Worst	2.39**E** + 01	1.59*E* + 02	2.92*E* + 01	9.35*E* + 01
		Mean	2.05**E** + 01	3.35*E* + 01	2.44*E* + 01	3.05*E* + 01
		Median	2.08**E** + 01	2.88*E* + 01	2.48*E* + 01	2.44*E* + 01
		StDev	2.21**E** + 00	3.42*E* + 01	2.50*E* + 00	1.70*E* + 01

*F*_5_	600000	Best	1.39*E* + 01	4.08*E* + 01	1.09**E** + 01	2.51*E* + 02
		Worst	3.28**E** + 01	1.78*E* + 02	3.38*E* + 01	3.72*E* + 02
		Mean	2.30*E* + 01	7.87*E* + 01	2.24**E** + 01	3.25*E* + 02
		Median	2.09**E** + 01	6.87*E* + 01	2.19*E* + 01	3.26*E* + 02
		StDev	5.33**E** + 00	3.53*E* + 01	6.87*E* + 00	2.99*E* + 01

*F*_6_	300000	Best	9.31*E* − 01	2.15*E* + 00	3.48**E** − 06	1.39*E* + 01
		Worst	2.41*E* + 00	3.92*E* + 00	2.01**E** + 00	1.61*E* + 01
		Mean	1.72*E* + 00	3.06*E* + 00	1.10**E** + 00	1.52*E* + 01
		Median	1.78*E* + 00	3.01*E* + 00	1.50**E** + 00	1.54*E* + 01
		StDev	4.01**E** − 01	6.64*E* − 01	7.73*E* − 01	5.90*E* − 01
